# Pediatric Anesthesia Exposure: Decoding Its Neurodevelopmental Implications and Navigating the Nuances

**DOI:** 10.7759/cureus.55952

**Published:** 2024-03-11

**Authors:** Wael AlKattan, Belal N Sabbah, Mohammad A Alghafees, Ahmad N Sabbah, Alanood Alsaleem, Meshari A Alqahtani, Alshaima Almadani, Aljazi Alrashid, Faris B Alshabanat, Mohammed S Ali Omar, Abderrahman Ouban, Mohamed Umair Aleem, Aladeen Barbour, Abdalrahman Abuzubida, Nadine A Osman, Saad S Ali, Zain Abbara, Mohammed A Alfuwais

**Affiliations:** 1 Surgery, Alfaisal University College of Medicine, Riyadh, SAU; 2 College of Medicine, Alfaisal University College of Medicine, Riyadh, SAU; 3 College of Medicine, King Saud Bin Abdulaziz University for Health Sciences, Riyadh, SAU; 4 Anesthesiology, King Faisal Specialist Hospital and Research Centre, Riyadh, SAU; 5 College of Medicine, Imam Mohammed Ibn Saud Islamic University, Riyadh, SAU; 6 Pathology, Alfaisal University College of Medicine, Riyadh, SAU

**Keywords:** pediatric exposure, neurodevelopmental conditions, anesthesia exposure, neurodevelopmental outcomes, pediatric anesthesia

## Abstract

General anesthesia is fundamental in pediatric medical interventions, but its potential neurodevelopmental impact on children has raised concerns, necessitating a thorough investigation. This systematic review aimed to assess the association between pediatric anesthesia exposure and neurodevelopmental outcomes, focusing on dosage effects and identifying high-risk groups. The study involved an extensive literature search across PubMed, Medline, and Google Scholar, selecting 40 relevant studies from an initial pool of 2,000, based on inclusion criteria that focused on children under 18 years exposed to anesthesia, excluding those with major comorbidities or perioperative physiological insults. It was observed that while a single exposure to anesthesia had minimal impact on general neurodevelopment, repeated or prolonged exposures posed greater concerns. Despite these findings, the study identified gaps in certain areas like adaptive behavior and sensory cognition due to limited data. The conclusion drawn is that although the evidence on anesthesia-induced neurotoxicity in children remains inconclusive, the implications of pediatric anesthesia exposure are significant enough to warrant careful consideration by healthcare professionals, who should balance the procedural benefits against the risks. This study also calls for future research to standardize methodologies and employ consistent, validated neurodevelopmental measurement tools.

## Introduction and background

Anesthesia plays an indispensable role in pediatric medical interventions. However, in recent years, there has been escalating apprehension regarding its potential implications on the neurodevelopmental trajectories of children [1]. The inherent vulnerability of the developing brain during childhood underscores the urgency of understanding the safety profile of anesthetic agents. While the necessity of anesthesia in surgical procedures is undeniable, the spotlight on its potential neurotoxic effects, especially as evidenced in animal studies, has intensified the research momentum in this domain.

A plethora of epidemiological endeavors have embarked on the mission to elucidate the relationship between early exposure to anesthesia and subsequent neurodevelopmental ramifications. Findings from both retrospective and prospective cohort studies suggest that early anesthesia (persons under the age of 18) exposure might be associated with a spectrum of cognitive challenges, encompassing learning disabilities, attentional deficits, and behavioral anomalies [2-4]. However, it's worth noting that based on current developmental assessment tools, a single exposure to general anesthesia does not appear to have a significant effect on general neurodevelopment, although prolonged or multiple anesthetic exposures may have some adverse effects [2].

The administration of anesthesia in pediatric surgical interventions is not just a medical necessity but also a potential variable influencing neurodevelopment. The heightened sensitivity of the brain during its formative years makes it particularly receptive to external agents, and the introduction of anesthesia during these pivotal phases could potentially sculpt long-term developmental pathways. This has catalyzed a surge in research endeavors, striving to decode the mechanisms underlying anesthesia-induced neurotoxicity and pinpoint potential risk factors [5,6]. Furthermore, recent clinical trials, including the Pediatric Anesthesia Neurodevelopment Assessment (PANDA) and Mayo Anesthesia Safety in Kids (MASK), have provided strong evidence that short exposure to general anesthesia at a young age does not result in detectable alterations in neurodevelopmental outcomes [7].

However, the complexity of the issue is highlighted by the fact that it remains challenging to discern whether any negative neurodevelopmental effects are due to the anesthetic drugs themselves, the conduct of anesthesia, surgical trauma, or the underlying clinical conditions that necessitated the surgery [8]. As the field continues to evolve, it is imperative for healthcare providers to remain updated on the latest findings and engage in proactive discussions to address potential misconceptions regarding the effects of general anesthesia on neurodevelopment in children [9]. Against this backdrop, our research delves into the critical inquiry of whether exposing children to general anesthesia compromises their neurodevelopment and if this potential risk is contingent upon the dose. Furthermore, we aim to discern if certain risk factors or specific vulnerable groups might be disproportionately susceptible to the possible adverse outcomes of anesthetic exposure.

## Review

Materials and methods

Inclusion and Exclusion Criteria

Studies were included if they focused on children younger than 18 years who had been exposed to anesthesia. Conversely, studies were excluded if they involved children who were unexposed to anesthesia, had major comorbidities, or had experienced a perioperative physiological insult.

Search Strategy and Data Extraction

**Figure 1:**
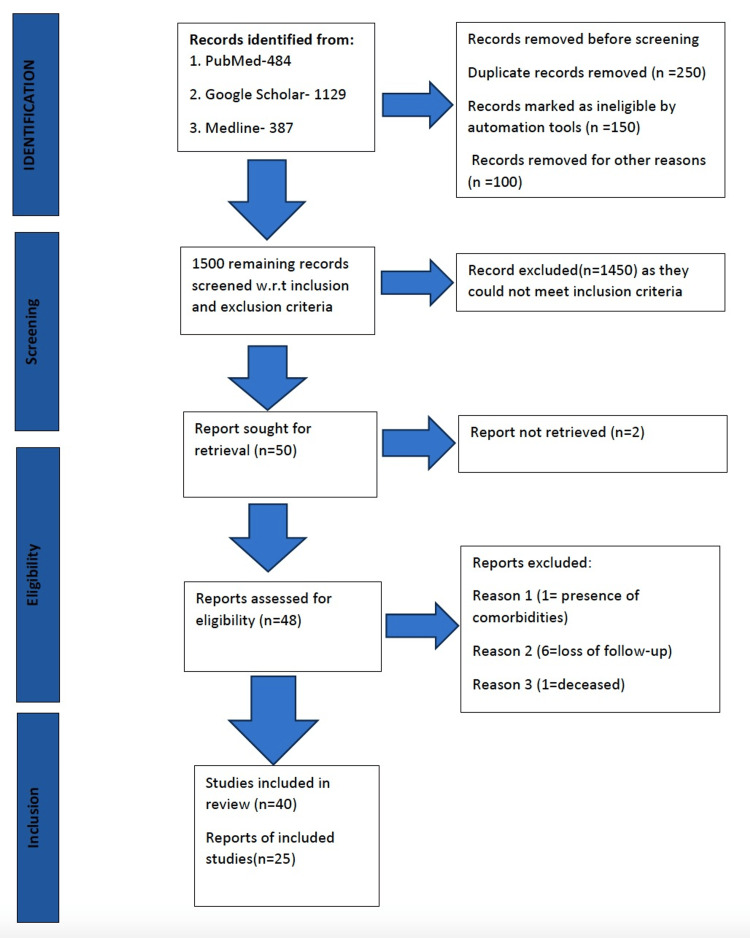
PRISMA flowchart diagram PRISMA: Preferred Reporting Items for Systematic Reviews and Meta-Analyses

A comprehensive literature search was executed across three primary databases: PubMed, Medline, and Google Scholar. For PubMed, search terms included combinations such as "Anesthesia Exposure OR Children", "Anesthesia Exposure AND Children", "Anesthesia Exposure AND Neurodevelopmental Outcomes", "Anesthesia Exposure in Children AND Neurodevelopmental Outcomes", "Pediatric Anesthesia AND Neurodevelopmental outcomes", "Anesthesia effects AND cognitive development", "Childhood Anesthesia AND neurotoxicity", and "Anesthetic Risk Factors AND neurodevelopmental outcomes". In Medline, the search was refined using terms like "Anesthesia Exposure AND Children", "Anesthesia Exposure AND Neurodevelopmental Outcomes", "Anesthesia Exposure in Children AND Neurodevelopmental Outcomes", "Anesthesia AND Vulnerable Groups in Children", and "Anesthesia AND childhood brain development". For Google Scholar, the primary search combination was "Anesthesia Exposure AND neurodevelopmental outcomes OR children". A detailed view of the search strategy can be seen in Table 1, and the PRISMA (Preferred Reporting Items for Systematic Reviews and Meta-Analyses) flowchart can be seen in Figure 1.

**Table 1:** Search strategy and data extraction

PubMed	Anesthesia exposure, children, neurodevelopmental system, neurodevelopmental outcomes	Anesthesia Exposure OR Children Anesthesia Exposure AND Children Anesthesia Exposure AND Neurodevelopmental Outcomes Anesthesia Exposure in Children AND Neurodevelopmental Outcomes Pediatric Anesthesia AND Neurodevelopmental outcomes Anesthesia effects AND cognitive development Childhood Anesthesia AND neurotoxicity Anesthetic Risk Factors AND neurodevelopmental outcomes	
Medline	Anesthesia exposure, children, neurodevelopmental system, neurodevelopmental outcomes	Anesthesia Exposure AND Children Anesthesia Exposure AND Neurodevelopmental Outcomes Anesthesia Exposure in Children AND Neurodevelopmental Outcomes Anesthesia AND Vulnerable Groups in Children Anesthesia AND childhood brain development	
Google Scholar	Anesthesia exposure, children, neurodevelopmental system, neurodevelopmental outcomes	Anesthesia Exposure AND neurodevelopmental outcomes OR children	

Data Collection

Upon identification of potential studies, a detailed review was undertaken. The full text of each article was assessed to extract relevant information, ensuring strict adherence to the inclusion criteria. Comprehensive data, encompassing essential participant details, intervention specifics, and trial outcomes, were extracted from the chosen articles and studies.

Results

In our systematic review, we extracted data from Google Scholar, PubMed, and Medline, focusing on children undergoing anesthesia procedures to evaluate potential neurodevelopmental outcomes.

The studies encompassed a patient age range of 0 to 18 years, with an even gender distribution: 50% female and 50% male. Our review spanned publications from 2017 to 2021. Out of the numerous studies reviewed, 40 met our inclusion criteria. These studies provided insights into various domains of neurodevelopmental derangements, such as adaptive behavior, cognition, behavioral issues, and the academic performance of the patients.

From our initial literature search, we identified 2,000 studies: 1,129 from Google Scholar, 387 from Medline, and 484 from PubMed. After deduplication, 1,500 unique studies remained. Screening by titles and abstracts led to the exclusion of 1,450 studies. After assessing the full texts of the remaining 50 studies for eligibility, 10 were further excluded, leaving 40 studies for review. A summary of the included studies can be found in Table 2.

**Table 2:** Summary of studies that evaluated different neurodevelopmental outcomes post-anesthesia exposure ADHD: Attention deficit hyperactive disorder, GA: General anesthesia, IQ: Intelligence quotient, PTSD: Post-traumatic stress disorder

Investigator	Age of exposure	Duration of exposure	Neurological symptoms	Developmental symptoms	Other symptoms
Kozanhan et al., 2018 [10]	7-12	Between 2 to 4 hours	No known	PTSD-related symptoms	State anxiety higher child post-traumatic stress reaction index
Tsai et al., 2018 [11]	Birth to 3 years	Multiple exposure each approximately ≥3 hours	ADHD	Not known	Not known
Warner et al., 2018 [12]	Birth to 3 years	Multiple exposures, analysis of single exposure vs. multiple exposure	Cognition derangements	PTSD	Slowness of movement
Banerjee et al., 2019 [13]	10 years to 18 years	Repeated exposure to general anesthesia	Neurocognitive impairments (not specified)	Not specified	Not specified
Khochfe et al., 2019 [14]	Before 2 years	Depends on duration of surgical procedures	Slowed learning	Difficulty walking	Not specified
Warner et al., 2019 [15]	Before 3 years	2 hours	Not specified	Not specified	Not specified
de Heer et al., 2017 [16]	0 to 5 years	Not specified	Reduction in non-verbal cognition	Non-verbal cognition impairment leads to developmental delays and altered apprehension	Not specified
Graham et al., 2016 [17]	0 to 4 years	Not specified (GA)	Poor neurodevelopmental outcomes	Deficits in development especially for children between 2 to 4 years. specifically, in the domains of communication, general knowledge, language, and cognitive domains	Not specified
Zaccariello et al., 2019 [18]	0 to 3 years	Not specified (GA).	Processing speed and fine motor skills associated with multiple (but not single) exposures to GA before age 3 years	Derangements in motor skills, visual-motor integration, and processing speed in children	Difficulty processing speed and motor skills
Niu et al., 2022 [19]	Postnatal year 2 or 3	Different durations	Neurodevelopmental impairment, cognitive dysfunction, and long-term adverse effects on neurocognitive outcomes	Processing speed, fine motor abilities, motor skills, visual-motor integration, and social and linguistic abilities	Neurotoxicity and developmental delays
DiMaggio et al., 2011 [4]	0 to 3 years	Varying durations	Anesthesia induced neurotoxicity	Not specified	Not specified
Ing et al., 2021 [20]	6 years	2 to 4 hours	Impaired memory	Language and communication problems	Not specified
Walkden et al., 2019 [21]	1 to 4 years	Multiple durations	Distorted executive functions	Developmental delays and difficulty in decision-making	Not specified
Wang et al., 2014 [3]	2 to 3 years	Not specified	Impaired learning, attention deficit hyperactive disorder	Distorted concentration	Not specified
Castellheim et al., 2018 [22]	13 to 21 years	Not specified (GA)	ADHD, learning disability	Autism Spectrum Disorder (ASD)	Congenital and systemic disorder
Kalman et al., 2009 [23]	0 – 6 years	4 hours	Apoptotic neuronal degeneration	Late cognitive impairment	Not specified
McCann et al., 2019 [24]	10 to 15 years	Abnormal development of CNS	Neurocognitive impairments	Not specified	Not specified
Vedovelli et al., 2019 [25]	6 to 12	1-3 hour	Decreased IQ	No significant changes	Not specified
Berghmans et al., 2009 [26]	18 to 30 months	1-2 hour	To some extend	Non-verbal reasoning and motor function	Not specified
Flick et al., 2009 [27]	0 to 4 years	No specific data provided	Learning disability and cognition	Academic performance	Behavioral changes

While our systematic review provided insights into several domains of neurodevelopmental outcomes, some domains could not be thoroughly evaluated due to a limited number of eligible resources. Specifically, domains such as adaptive behaviors, general health, sensory, and social cognition remained underexplored in the context of anesthesia exposure.

Consistent with prior research, our findings suggest that anesthesia exposure at a young age is associated with neurocognitive deficits. However, it's imperative to note that the clinical evidence supporting anesthesia-induced neurotoxicity remains limited. Despite the inconclusive nature of the evidence, the potential implications of anesthesia exposure at a young age cannot be overlooked. Given the clinical ramifications, healthcare practitioners must exercise prudence when considering the necessity of anesthesia exposure in children, weighing the potential risks against the benefits of the procedure.

Discussion

Our systematic review suggests that a single exposure to general anesthesia does not significantly impact general neurodevelopment. While numerous studies have indicated that anesthesia can influence various developmental domains at different ages, the evidence predominantly suggests that the detectable impact is typically minor. Consequently, these concerns should not deter essential medical procedures.

However, when patients might require multiple procedures, this becomes a more pressing consideration. Repeated or prolonged anesthetic exposures could have more pronounced detrimental effects on neurodevelopment. For instance, a study in 2017 found that general anesthesia affected IQ levels up to three months post-exposure [28]. A meta-analysis in 2018 supported the hypothesis of an association between the age of anesthesia exposure and subsequent neurodevelopmental deficits [12]. Another cohort study in the same year documented a link between attention deficit and hyperactivity disorder and anesthesia exposure [11]

The intricate nature of anesthetic planning and execution for pediatric patients underscores the importance of involving a pediatric anesthesiologist. Their expertise is crucial, given the complexities and nuances of various anesthetic techniques, patients, and surgical procedures. Research has also delved into the effects of specific anesthetic agents. For instance, a study highlighted the adverse effects of volatile anesthetic agents on neurodevelopment [29]. Another retrospective cohort study aimed to find an association between perioperative anesthesia exposure and neurodevelopmental outcomes in 12-month-old neonates. After accounting for multiple relevant covariates, they found diminished neurodevelopment in the targeted population [30].

Recent studies have further elucidated these findings. For example, the general anesthesia spinal (GAS), PANDA, and MASK studies provided robust evidence that short exposure to general anesthesia at a young age does not result in detectable alterations in neurodevelopmental outcomes [7]. Another study emphasized that children exposed to anesthesia before two years of age have an increased risk of developmental delay, which is further elevated with increased frequency of anesthesia and longer total anesthesia duration [31].

Limitations

This systematic review has several limitations. Notably, it lacks formal bias analysis, which is essential for assessing publication bias. The studies included exhibit heterogeneity in design, demographics, and outcome measures, potentially affecting the generalizability of the findings. Variability in anesthetic regimens and exposure duration across studies adds complexity, and most studies provide short-term rather than long-term follow-up data. Additionally, the possibility of unmeasured confounding variables influencing the results cannot be ruled out. Despite these limitations, our findings contribute valuable insights into the neurodevelopmental implications of pediatric anesthesia exposure, while highlighting areas needing further research.

## Conclusions

The current literature does have identifiable limitations that could guide the design of future research. A myriad of techniques were used to measure neurodevelopmental outcomes. Upcoming clinical research endeavors should focus on standardizing patient and anesthetic characteristics, using validated neurodevelopmental measurement tools at consistent stages in a child's development, and collecting data prospectively.
